# Immunization with recombinant enolase of *Sporothrix* spp. (rSsEno) confers effective protection against sporotrichosis in mice

**DOI:** 10.1038/s41598-019-53135-z

**Published:** 2019-11-20

**Authors:** Deivys Leandro Portuondo, Paulo Roberto Dores-Silva, Lucas Souza Ferreira, Carlos S. de Oliveira, Damiana Téllez-Martínez, Caroline Maria Marcos, Maria Luiza de Aguiar Loesch, Fanny Guzmán, Lisandra M. Gava, Júlio César Borges, Sandro Antonio Pereira, Alexander Batista-Duharte, Iracilda Zeppone Carlos

**Affiliations:** 1 São Paulo State University (UNESP), School of Pharmaceutical Sciences, Department of Clinical Analysis, Araraquara, SP Brazil; 2 São Carlos Institute of Chemistry, University of São Paulo, São Carlos, SP P.O. Box 780, 13560–970 Brazil; 3 Núcleo Biotecnológico de Curauma (NBC), Pontificia Universidad Católica de Valparaíso, Valparaíso, Chile; 4 Center of Biological and Health Sciences, Federal University of São Carlos, São Carlos, SP ZIP Code 13560-970 Brazil; 5 Oswaldo Cruz Foundation (Fiocruz), Evandro Chagas National Institute of Infectious Diseases (INI), Laboratory of Clinical Research on Dermatozoonoses in Domestic Animals (Lapclin-Dermzoo), Rio de Janeiro, RJ Brazil

**Keywords:** Protein vaccines, Protein vaccines, Infection, Infection

## Abstract

In recent years, research has focused on the immunoreactive components of the *Sporothrix schenckii* cell wall that can be relevant targets for preventive and therapeutic vaccines against sporotrichosis, an emergent worldwide mycosis. In a previous study, we identified a 47-kDa enolase as an immunodominant antigen in mice vaccinated with an adjuvanted mixture of *S. schenckii* cell wall proteins. Here, we sought to assess the protective potential of a *Sporothrix* spp. recombinant enolase (rSsEno) formulated with or without the adjuvant Montanide Pet-GelA (PGA) against the *S. brasiliensis* infection in mice. Mice that were immunized with rSsEno plus PGA showed increased antibody titters against rSsEno and increased median survival time when challenged with *S. brasiliensis* as compared with mice that had not been immunized or that were immunized with rSsEno alone. Immunization with rSsEno plus PGA induced a predominantly T-helper 1 cytokine pattern after *in vitro* stimulation of splenic cells with rSsEno: elevated levels of IFN-γ and IL-2, as well as of other cytokines involved in host defense against sporotrichosis, such as TNF-alpha, IL-6, and IL-4. Furthermore, we show for the first time the presence of enolase in the cell wall of both *S. schenckii* and *S. brasiliensis*. As a whole, our results suggest that enolase could be used as a potential antigenic target for vaccinal purposes against sporotrichosis.

## Introduction

Sporotrichosis is a subcutaneous mycosis of subacute or chronic evolution mainly caused by traumatic inoculation of different species of the *Sporothrix* genus affecting both humans and animals^1^. The disease has a universal geographical distribution, although it is endemic in Latin America, including in Peru, México, Colombia, Guatemala and, especially, Brazil, where in the last 20 years, it became an important zoonosis, with the infected cat being the main source of transmission^2–4^. Species of the *Sporothrix* genus are thermodymorphic fungi with a saprophytic life at 25 °C and a filamentous form. The parasitic form at 35–37 °C is a yeast^1,5^. The human infection is acquired in two ways: traumatic inoculation through the skin with *Sporothrix* species (spp.) or inhalation. Zoonotic transmission principally occurs from infected cats to humans^6^.

The genus *Sporothrix* is currently classified into two clades: i) the clinical clade, which includes *S. brasiliensis*, *S. globosa*, *S. luriei* and *S. schenckii* sensu stricto and ii) the environmental clade, composed mainly of species less pathogenic to man and animals, such as *S. mexicana*, *S. pallida* and *S. chilensis*^7,8^. Brazil is the only country that has reported all species of the clinical clade, and *S. brasiliensis* is the most virulent species^9,10^. This species is also the most prevalent during zoonotic transmission through scratches and bites from infected cats^8^. In this country, though sporotrichosis has been reported in most states, the disease is a neglected disease, particularly in the state of Rio de Janeiro, where the largest number of cases has been reported, representing a serious public health problem^3^. The Oswaldo Cruz Foundation (Fiocruz), Rio de Janeiro, a referral center for the diagnosis and treatment of this mycosis, diagnosed over 4000 humans and feline sporotrichosis cases between 1998 and 2012^11^. More recently, according to data from the epidemiological bulletin of 001/2018 of the sanitary vigilance service of the state of Rio de Janeiro, from January 2015 through May 2018, more 3510 human cases were confirmed^12^, which shows a progressive increase in the incidence and prevalence of this mycosis.

Sporotrichosis is usually controlled through the use of itraconazole – in combination with potassium iodide in cats –, or terbinafine in immunocompetent patients who exhibit the less severe clinical forms of the disease (lymphocutaneous and fixed cutaneous lesions)^13,14^. However, in immunocompromised patients with neoplastic diseases, transplantation or AIDS, the conventional treatment with classical antifungals is generally ineffective^15,16^. The lack of a veterinary and/or human vaccine against this disease has awakened interest in the identification of *S. schenckii* cell wall immunoreactive components involved in fungal pathogenesis^17^ and the induction of the immune response^18^ that can be used for immunoprophylaxis and immunotherapy against sporotrichosis.

In previous studies, our group showed that sera obtained from mice immunized with an *S. schenckii*- cell wall protein (CWP) formulated with the adjuvant aluminum hydroxide (AH) showed reactivity against two proteins, one of 71 kDa and another of 47 kDa. The latter was functionally identified as enolase and predicted to be an adhesin by the Fungal RV database^19^. These immune sera showed opsonizing properties, enhancing the phagocytosis of *S. schenckii*, and they inhibited the fungal adhesion to fibroblasts *in vitro*. Passive transfer of immune serum to nonimmunized mice conferred protection against challenges with the fungus. These findings indicated the induction of protective immunity from the vaccine formulation against experimental sporotrichosis and the potential use of both antigens for an antifungal vaccine. More recently, we showed that serum from mice vaccinated with AH-adsorbed CWPs, and serum obtained from mice immunized with the same antigenic source but formulated with Montanide Gel Pet A adjuvant (PGA), reacted with the *S. brasiliensis* yeast cell wall^20^. Such cross-reactivity, as well as the fact that both formulations confer protection in mice challenged either with *S. schenckii or S. brasileinsis*, suggested the existence of shared immunodominant antigens that could prove beneficial for the simultaneous protection against these species, which are the more virulent of the genus *Sporothrix*.

Enolase (2-phospho-D-glycerate hydrolase, EC 4.2.1.11) is a metalloenzyme that requires the metal ion magnesium (Mg^2+^) to catalyze the dehydration of 2-phosphoglycerate (2-PG) to phosphoenolpyruvate (PEP), a product that is used to produce energy (ATP) in eukaryotic and prokaryotic cells^21^. In mammals, there are at least 4 isoforms of enolase: α‐enolase (eno1), expressed in almost all tissues; β‐enolase (eno3), predominantly expressed in adult skeletal muscle; γ‐enolase (eno2), found in neurons and neuroendocrine tissues^22^; and eno4, expressed in human and mouse sperm^23^. Enolase has been identified on the cell surface of *C. albicans*^24^, *Plasmodium falciparum*^25^, *Ascaris suum*^26^, *Streptococcus sobrinus*^27^, *S. suis serotipo* II^28^, S*. iniae*^29^, *Plasmodium spp.*^30^ and *Clonorchis sinensis*^31^. In addition, the immunogenicity and protective properties of anti-enolase immune response have been reported for diverse pathogens^24,27,32^. Furthermore, enolase is probably the only immunogenic antigen shared by eukaryotic and prokaryotic pathogens that has been proposed as an antigenic target for diagnostic, therapeutic, and prophylactic purposes against different diseases.

In this study, we report for the first time the presence of enolase in the cell wall of *Sporothrix* spp. and also that a PGA-adjuvanted vaccine formulation using a recombinant enolase was able to confer protection either actively to a subsequent challenge with *S. brasiliensis* or passively through the serum of vaccinated mice. Our results thus suggest that enolase could be used as a potential antigenic target for vaccinal purposes against sporotrichosis.

## Results

### Production, purification, and characterization of rSsEno

**Figure 1:**
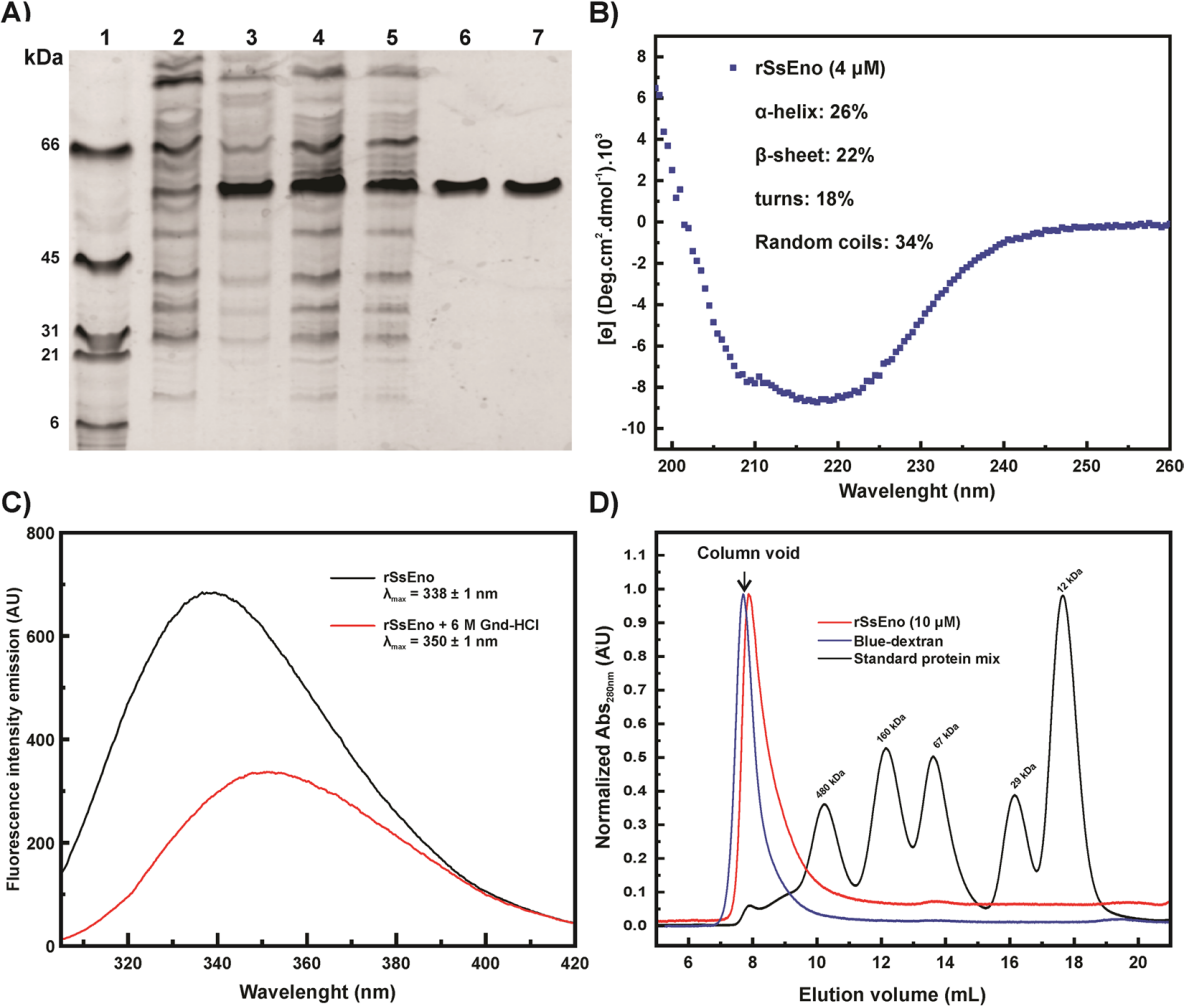
SDS-PAGE and structure analysis of rSsEno expressed in *E. coli* BL21. The recombinant plasmid pET28a::SsEno-transformed *E. coli* BL21 cells were induced in the presence of 0,2 mM IPTG for 4 h at 30 °C. The cells were lysed by sonication, and the supernatant containing the recombinant protein was purified by affinity and preparative SEC, respectively. All the samples were analyzed by SDS-PAGE 12%, and the protein was stained with Coomassie Blue R250 in the gel. (**A**) Expression and purification of rSsEno. Molecular mass markers in kDa (lane 1), non-IPTG-induced pET28a::SsEno-transformed *E. coli* BL21 cells lysate (lane 2), IPTG-induced pET28a::SsEno-transformed *E. coli* BL21 cells lysate (lane 3); supernatant of lysed IPTG-induced pET28a::SsEno-transformed*E. coli* BL21 cells (lane 4), supernatant of lysed cells filtered through Hydrophlic Durapore Membrane, 0.45 µm cutoff, 47 mm diameter (lane 5), rSsEno purified by Ni^2+^ affinity chromatography (lane 6) and by preparative SEC (lane 7). (**B**) The CD spectrum shows that rSsEno was obtained mainly with a secondary structure composed by α-helices and β-sheets. (**C**) Intrinsic emission fluorescence spectra for enolase in the folded (black curve) and unfolded (red curve) states induced by 6 M Gnd-HCl. (**D**) Analytical SEC performed for rSsEno (red line). The MW standard protein mix elution pattern is represented by the black line: 1) Apoferritin (480 kDa); 2) γ-Globulin (160 kDa); 3) BSA (67 kDa); 4) carbonic anhydrase (29 kDa); 5) Cytochrome C (12 kDa). The column void is identified by blue dextran (blue line).

Figure 1A shows that rSsEno expressed in the IPTG-induced pET28a::SsEno-transformed *E. coli *BL21 cells was produced both in the pellet, as well as in soluble fraction of the lysed cells (Fig. 1A, lane 3). Based on this result, the rSsEno containing the soluble fraction (after filtration, Fig. 1, lane) was purified by Ni^2+^-affinity (Fig. 1A, lane 6) and preparative SEC (Fig. 1, lane 7), respectively, resulting in an apparent purity 95% on SDS- PAGE with Coomassie blue staining. The final yield was approximately of 15 mg of pure rSsEno per L.

The rSsEno far UV-CD spectrum shows two bands at ~209 and 218 nm, which indicate the presence α-helices and β-sheets folded structures (Fig. 1B). Deconvolution analysis of the rSsEno far UV CD spectra revealed that the secondary structure of the proteins in the preparation contains 24% α-helices, 22% β-sheets, 18% of turns and 34% of random coils. Intrinsic emission fluorescence also indicated that the rSsEno was purified in a folded state. The mean maximum emission emission wavelength (λ_max_) of about 338 nm indicates that the 6 tryptophan residues present in the rSsEno structure were partially protected from the solvent (Fig. 1C). On the other hand, treatment of rSsEno with a chemical denaturant caused a red shift and fluorescence quenching suggesting at least a partial exposition of the tryptophan residues to the solvent^33^. Analytical SEC analysis showed that rSsEno elutes near the column void (identified by blue dextran in Fig. 1D) leaving a tail after the main peak, which indicates the protein sample as a heterogeneous solution probably due to the presence of oligomers of smaller size in equilibrium; this suggests that the recombinant protein should be organized by various oligomeric forms. Small angle x-ray scattering (SAXS) data (not shown) indicated that rSsEno behaved as an oligomeric particle with a weight-average molecular weight of 580 ± 60 kDa. Taken together, our biophysical data suggest that rSsEno was purified folded and as a mixture of different oligomeric forms.

### Sequence alignment of the *S. schenckii* enolase

**Figure 2:**
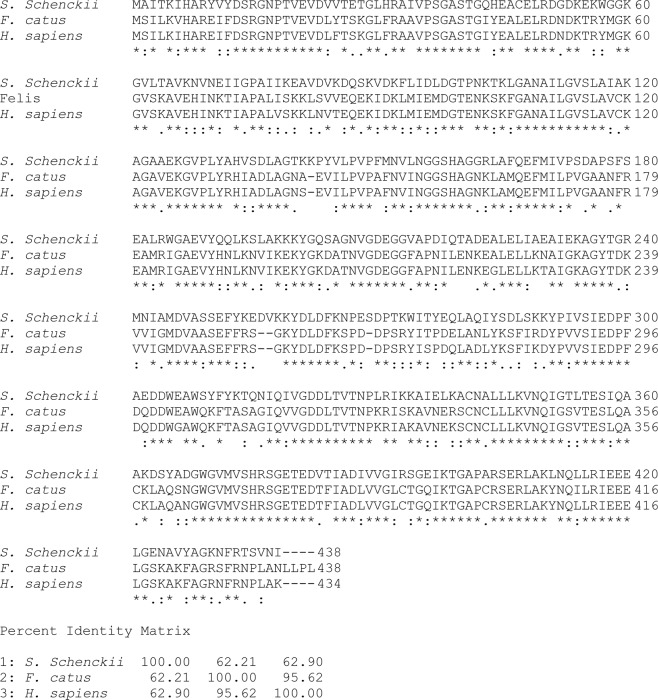
Multiple sequence alignments of *S. schenckii*. The deduced amino acid sequence of *S. schenckii* (ERS97971.1), *Felis catus* (M3WCP0_FELCA) and *Homo sapiens* (P06733) were aligned by the Clustal Omega server. The conserved amino acids in all sequences are labeled with asterisks; the conservative and semi-conservative substitutions are labeled with two and one points, respectively. The percentage of amino acid sequence identity between all enolases is indicated.

The sequence alignment analysis among the *S. schenckii-, F. catus-* and *H. sapiens*-enolase revealed an expected result; the enolase from humans and cats showed a degree of identify of 95% (Fig. 2). However, both enolases showed an identity of 62% with the enolase of *S. schenckii*. Moreover, although we don’t present it in alignment form, the *S. schenckii* enolase has 100% sequence identity with the ones from *S. schenckii* 1099–18 and *S. brasiliensis* 5110, belonging together to the Cluster identity UniRef100 U7PSS1. Specifically, according to the uniprot database (https://www.uniprot.org/), there are five enolase genes in the *Sporothrix* spp. genome, of which three, namely SPSK_03292, HMPREF1624_06143, and SPBR_00513, code for the same 438 a.a. protein in *S. schenckii* ATCC58251, *S. schenckii* 1099–18, and *S. brasiliensis* 5110, respectively; the two other genes, namely SPI_04152 and SPI_06529, encode the 439 and 465 a.a. proteins found in *S. insectorum* RCEF 264. Unlike *S. schenckii* and *S. brasiliensis*, *S. insectorum*, is not clinically associated with human and feline sporotrichosis.

### Specificity of the anti-rSsEno serum

**Figure 3:**
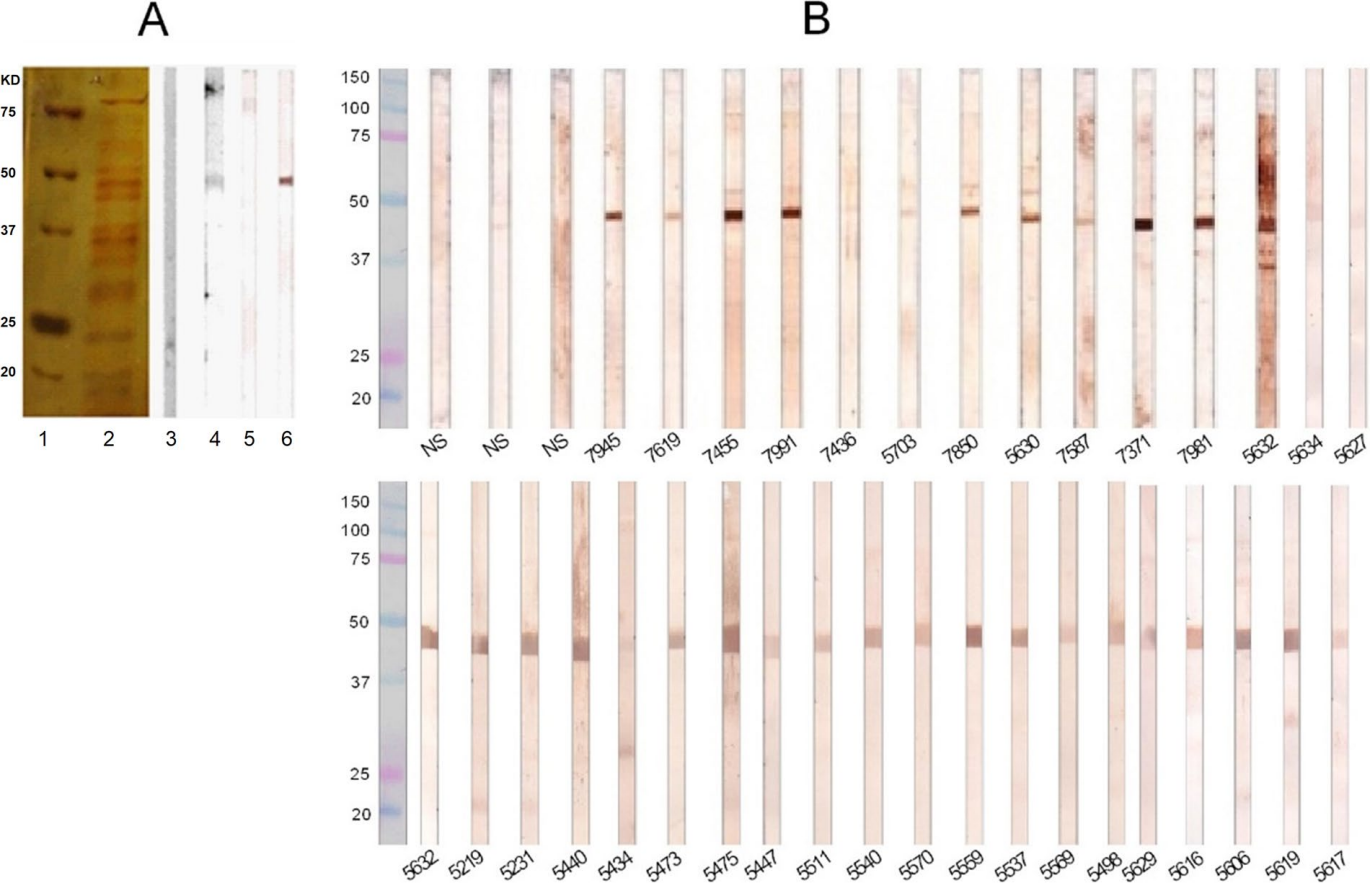
Western blot analysis showing the specificity of the anti-rSsEno sera and the reactivity of the sera from cats with sporothricosis against rSsEno. Samples of *S. schenckii* ATCC 16345 CWPs and rSsEno were tested by 12% SDS-PAGE under nonreducing conditions and after immobilization on a nitrocellulose membrane. The strips were incubated at 37 °C for 1 h with anti-rSsEno serum or naïve mouse serum, and the immunoblots were visualized by adding 3,3′-diaminobenzidine substrates after being treated with goat anti-mouse IgG-HRP. Panel A, column 1: molecular weight marker; column 2: *S. schenckii* ATCC 16345 CWPs resolved by SDS-PAGE 12%; columns 3 and 4: nitrocellulose strips containing the *S. schenckii* ATCC 16345 CWPs treated with NS and anti-rSsEno serum, respectively; columns 5 and 6: nitrocellulose strips containing rSsEno treated with naïve mice-serum and anti-rSsEno serum, respectively. Panel B, strips containing rSsEno were incubated with sera from cats with or without sporotrichosis (NS) and immunoblots were incubated with goat anti-feline IgG-HRP. Each cat serum is identified by the admission number of the Laboratory of Clinical Research in Dermatozoonoses in Domestic Animals of the National Institute of Infectology Evandro Chagas (FIOCRUZ).

The specificity of the antibodies raised in rSsEno immunized mice was examined by immunoblotting against recombinant enolase or CWPs isolated from *S. schenckii* ATCC 16345. As shown in Fig. 3 (A), the anti-rSsEno sera reacted with the recombinant protein and against a single reactive band present in *S. schenckii* ATCC 16345 CWPs with the expected 47 kDa molecular mass, corresponding to the native enolase^19^. Interestingly, the serum obtained from infected cats with sporotrichosis confirmed a specific high reactivity against the recombinant protein (Fig. 3B,C), indicating that, during natural infection, the fungal enolase can induce anti-enolase antibodies. Sera from uninfected control cats exhibited no immunoreactivity with the rSsEno (Fig. 3B).

### Enolase is present in the cell wall of *S. schenckii* spp

**Figure 4:**
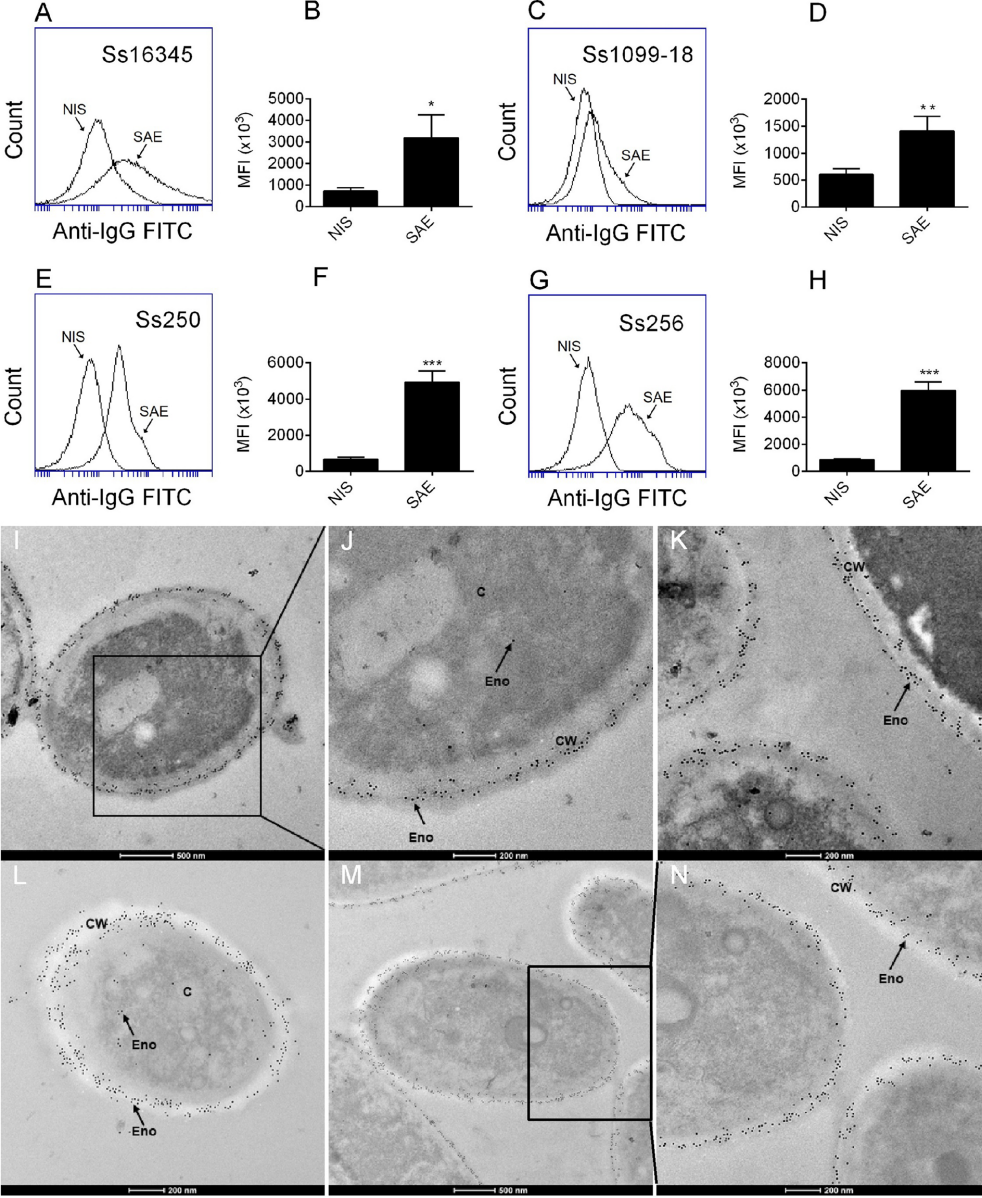
Demonstration of the enolase on the cell surface of *Sporothrix spp.-*yeasts by flow cytometry and electron microscopy. A *S. schenckii* ATCC 16345 (Ss16345), *S. schenckii* 1099–18 (Ss1099–18), *S. brasiliensis* Ss250 (Ss250), or *S. brasiliensis* Ss256 (Ss256) yeasts suspension was previously incubated with anti-rSsEno serum (SAE) or serum from nonimmunized mice (NIS) for 1 h at 37 °C. After washing, the cells were exposed to FITC-conjugated rabbit anti-mouse IgG and examined using a flow cytometer. (**A**,**C**,**E**,**G**) Representative histograms from one of three independent experiments for the indicated *Sporothrix* spp. yeasts treated with NIS or SAE. Bar graphs show the median fluorescence intensity (MFI) of the FITC staining for *S. schenckii* ATCC 16345 (**B**), *S. schenckii* 1099–18 (**D**), *S. brasiliensis* Ss250 (**F**) and *S. brasiliensis* Ss256 (**H**). The results are presented as the mean ± SD of three independent experiments, and statistical significance was determined by Student’s paired *t* test (I-N). *P < 0.05; **P < 0.01 ***P < 0.001. Ultrathin sections of each fungus were incubated overnight with SAE or NIS and then treated overnight with an Au-conjugated secondary antibody, at 4 °C. Grids were observed with in a transmission electron microscope after being stained with uranyl acetate and lead citrate. The micrographs show enolase (Eno) on the cell wall (CW) or cytoplasm (**C**) of *S. schenckii* ATCC 16345-(**I,J**), *S. schenckii* 1099–18 (**K**), or *S. brasiliensis* Ss250 (**L**) yeasts treated with SAE. (**M,N**) show Eno on the CW of *S. brasiliensis* Ss256-yeasts treated with SAE.

After confirming their specificity, the anti-rSsEno serum was used to detect enolase in the *S. schenckii* ATCC 16345, *S. schenckii* 1099–18, *S. brasiliensis* Ss250 and *S. brasiliensis* Ss256 cell wall. Figure 4(A,C,E,G) shows an intense and significant (p < 0.05) median fluorescence intensity (MFI) in yeasts treated with the anti-rSsEno serum compared to yeast treated with serum from nonimmunized mice (NIS), evidencing enolase on the cell surface of these strains. The MFI shift was greater for *S. brasiliensis* Ss250 and Ss256 (Fig. 4F–H) compared to *S. schenckii* ATCC 16345 (Fig. 4B) and *S. schenckii* 1099–18 (Fig. 4D), suggesting that this protein is expressed more on the cell wall of *S. brasiliensis*, the more virulent species. The presence of enolase on the cell surface of the studied fungi was also confirmed by transmission microscopy using the immunogold stain. Figure 4(I–N) showed that enolase appears distributed along the cell wall of *S. schenckii* ATCC 16345, *S. schenckii* 1099–18, *S. brasiliensis* Ss250 and *S. brasiliensis* Ss256, which might facilitate its recognition by the host’s immune system, although it also appears, as expected, in the cellular cytoplasm of these species, since its classical function is to catalyze the reversible conversion of 2-phosphoglycerate to phosphoenolpyruvate^22,34^.

### Antibody response

**Figure 5:**
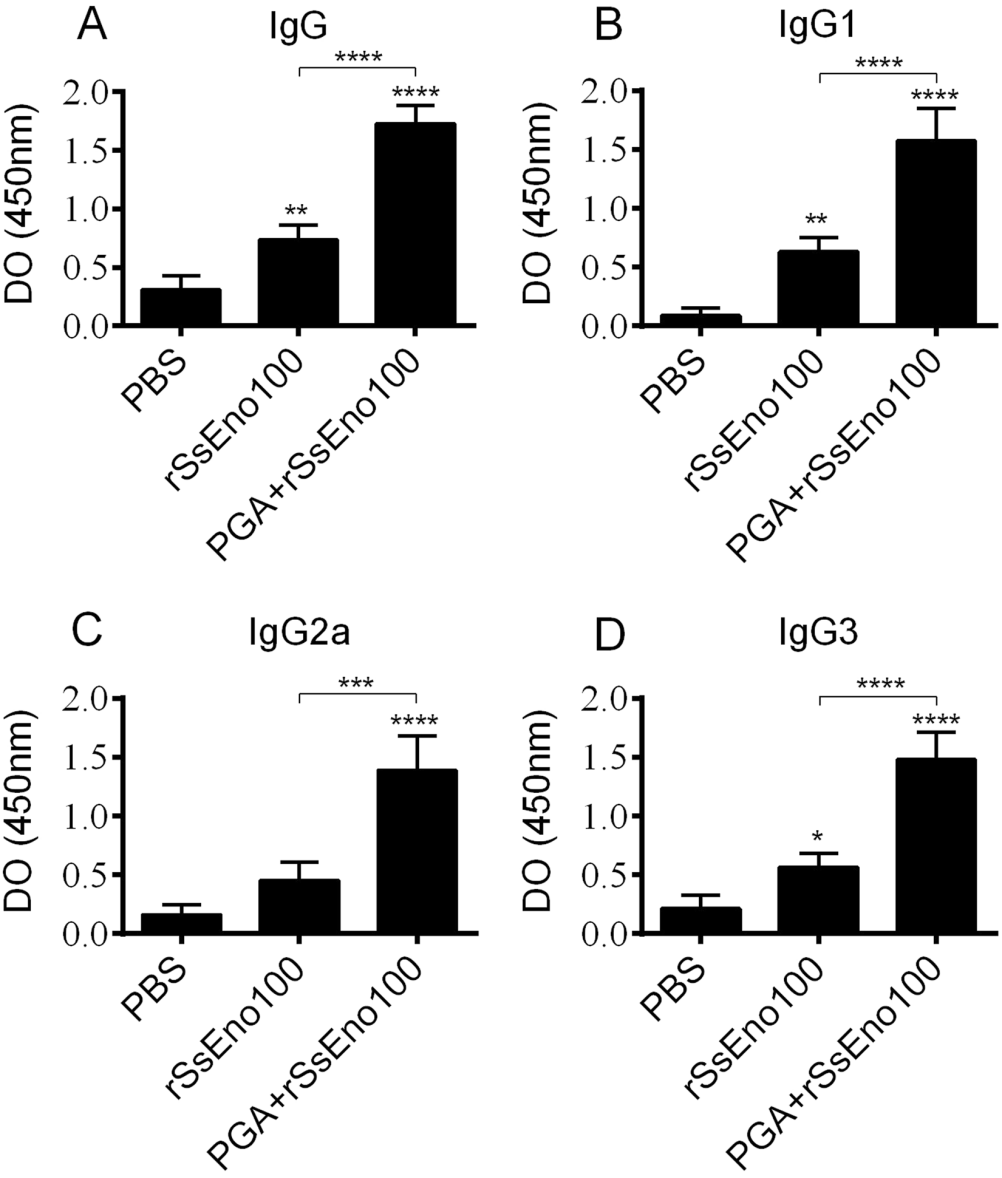
Immunization with rSsEno with or without PGA conjugation enhanced the antibody response. BALB/c mice were s.c. immunized three times with rSsEno100, PGA + rSsEno100 or PBS as a negative control. Sera collected seven days after the last boost was used to determine antigen-specific IgG (**A**), IgG1 (**B**), IgG2a (**C**), and IgG3 (**D**) titers by ELISA. The results are presented as the mean ± SD of 5 mice from one of three independent experiments, and statistical significance was determined by one-way ANOVA using Tukey’s multiple comparisons test and a 95% confidence interval. *(p < 0.05), **(p < 0.01), ***(p < 0.001) and ****(p < 0.0001) for comparison with the control group or as indicated.

To assess the immunogenic potential of *S. schenckii*-enolase, sera from the experimental group obtained seven days after the last boost was subjected to ELISA using rSsEno as an antigen. Our results showed that animals immunized only with enolase stimulated high IgG specific antibody production (Fig. 5A) compared to the PBS group. However, as expected, the specific antibody production was significantly higher (p < 0.05) when enolase was formulated with the PGA adjuvant. We also determined the rSsEno-specific IgG1, IgG2a and IgG3 antibodies induced by each formulation. Mice immunized with rSsEno100 and PGA + rSsEno100 induced higher IgG1 and IgG3 antibody levels against rSsEno compared to the PBS control group, but the level of both subclasses was higher in the mice immunized with the PGA-adjuvanted formulation (Fig. 5B,C). The PGA + rSsEno100 formulation was the only formulation that induced the production of IgG2a (Fig. 5C).

### Cytokine profile analysis

**Figure 6:**
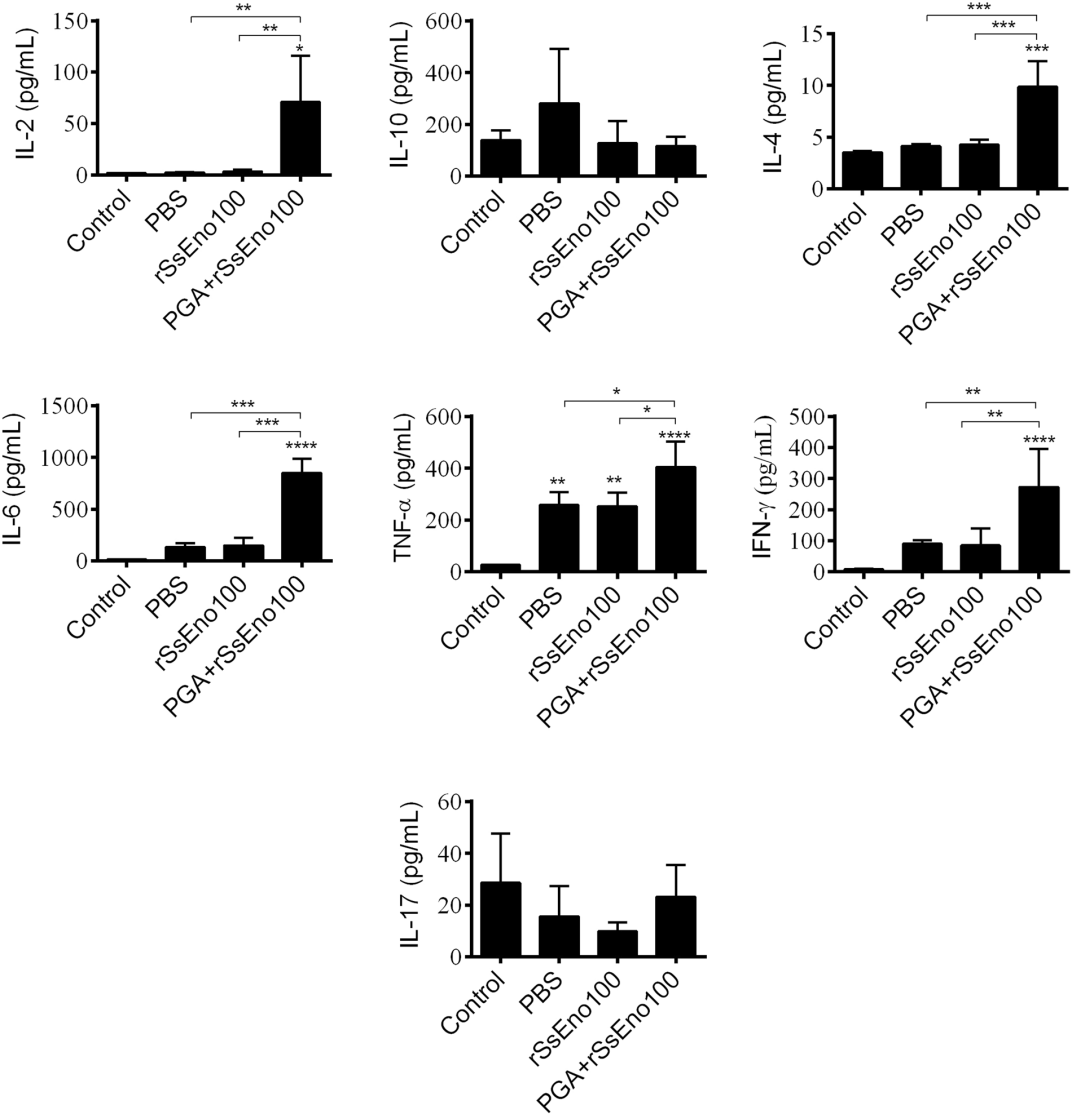
Vaccinated mice with rSsEno100 and PGA + rSsEno100 showed differences in Th1, Th2 and Th17 cytokine profiles. BALB/c mice were s.c. immunized three times with rSsEno100, PGA + rSsEno100 or PBS as a negative control. Total splenocytes of each animal were obtaining seven days after the last immunization and stimulated *in vitro* with rSsEno. After 24 h of incubation, supernatant-accumulated cytokines (IL-2, IL-4, IL-6, IL17A, IFN-γ, TNF and IL-10) were measured by cytokine cytometric bead array kit ELISA. The results are presented as the mean ± SD of 5 mice from one of three independent experiments, and statistical significance was determined by one-way ANOVA using Tukey’s multiple comparisons test and a 95% confidence interval. *(p < 0.05), **(p < 0.01), ***(p < 0.001) and ****(p < 0.0001) for comparison the control group or as indicated.

The effect of anti-enolase vaccination on the pattern of cytokines was evaluated in the supernatant of splenocyte cultures from nonimmunized and immunized mice after *in vitro* stimulation with rSsEno100. A higher production of IL-2 and IFN-ɣ from the Th1 profile, IL-4 and IL-6, which are involved in the production of antibodies, and TNF-α, which is released during the innate immune response (also with IL-6) in mice vaccinated with PGA + rSsEno100, was observed (Fig. 6). All of these cytokines are involved in defense against *S. schenckii*, which is additional evidence of protective immunogenicity induced by the vaccine formulation.

### Challenge studies

**Figure 7:**
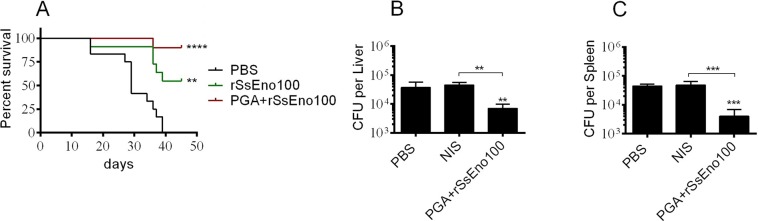
rSsEno immunization induces protection against disseminated sporotrichosis. BALB/c mice were immunized (s.c.) three times with rSsEno100, PGA + rSsEno100, or PBS and after seven days and after seven days were challenged i.v. with 1 × 10^5^ *S. brasiliensis* Ss250 yeast cells. Mice survival was monitored daily for 45 days post-challenge (n = 10 in all groups). In another study, two hours prior to infection with 10^6^ *S. brasiliensis* Ss250 yeasts, BALB/c mice were passively immunized (i.p.) with a pool of sera from PGA + rSsEno100-, NIS- (serum from non-immunized mice), or PBS-injected mice; five days post-infection the number of CFUs was determined in the spleen (**B**) and liver (**C**) of each animal. Differences in survival were determined by the log-rank test. Results of the passive immunization study are presented as the mean ± SD of 7 mice from one of two separate experiments. Statistical significance was determined by one-way ANOVA using Tukey’s multiple comparisons test and a 95% confidence interval. **(p < 0.01), ***(p < 0.001), and ****(p < 0.0001) for comparisons with the control group or as indicated.

To test whether rSsEno in the formulation with PGA adjuvant protects against systemic sporotrichosis in mice, seven days after booster immunization, mice from each group were challenged intravenously with 10^5^ *S. brasiliensis* Ss250 yeasts, a highly virulent strain. The mortality of nonimmunized mice was of 100% before 40 days postinfection, while the rSsEno-immunized mice showed over 50% survival, and those immunized with the PGA-adjuvanted formulation exhibited the highest percentage of survival (over 90%) at the end of the experiment (45 days postinfection) (Fig. 7A). Furthermore, passively immunizing mice with anti-rSsEno serum led to a significant decrease in the number of CFUs in the spleen (P < 0.01) and liver (p < 0.001) following infection with *S. brasiliensis* Ss250 via the i.p. route as compared to those previously treated with NIS or PBS. Our results thus indicate that enolase has potential use as an immunogen for both therapeutic and prophylactic purposes.

## Discussion

In the last two decades, sporotrichosis has been a hyperendemic zoonosis in Brazil transmitted by infected cats. The high incidence of sporotrichosis, together with the low effectiveness of the treatment, especially in immunocompromised individuals, has reinforced the need to identify antigenic targets on the cell surface of species of clinical interest of the genus *Sporothrix* for immunological prevention and therapeutic intervention^17,35^.

In this study, the enolase of *S. schenckii* was obtained by expression in *E. coli*, then purified and partially characterized. Our results showed that the His-tagged rSsEno was successfully produced with a molecular weight of 50 kDa and with a native-like structure as suggested by signals of the presence of secondary and local tertiary structures, obtained in the CD and tryptophan fluorescence experiments. We expected that rSsEno would be assembled in the form of dimers, as reported for yeast enolase^36,37^. However, analytical SEC indicated the presence of various oligomeric species of a weight-average molecular weight of about 580 kDa as indicated by SAXS data.

Enolase has been described as a moonlighting protein that exhibits multiple nonglycolytic functions, probably because of its different multimeric structures^32^. Ehinger *et al*.^38^ reported that α-enolase of *Streptococcus pneumonia* forms an octamer in solution and that due to its binding to human plasminogen, it probably resides on the cellular surface of this pathogen and can be involved in virulence. Wu *et al*.^39^ also reported that *Staphylococcus aureus* recombinant enolase is organized in dimers and octamers and that the latter probably exist *in vivo* since it showed enzymatic activity *in vitro*. Whether the complex oligomeric state of rSsEno in solution is the same as its native form in *S. schenckii*, and its functional role *in vivo*, is a subject for future studies.

The reactivity of sera from cats with sporotrichosis against rSsEno and the lack of reactivity with sera from uninfected control cats evidenced the antigenic role and probable immunogenicity of the *S. schenckii* enolase during the infectious process in these animals. Coupled with a 38% difference in sequence homology, this indicates that *S. schenckii* enolase may contain conserved regions distinct from its cat and human orthologs, suggesting that *S. schenckii* enolase can be used for vaccine and/or therapeutic strategies against or as a diagnostic tool for sporotrichosis in cats. Although α-enolase autoantibodies have been associated with a wide variety of human autoimmune diseases including systemic lupus erythematosus, autoimmune-mediated retinopathy, autoimmune hepatitis, severe asthma, and Hashimoto’s encephalopathy^22^, as far as we know, the association of these antibodies with clinical autoimmune disorders in cats is unknown. In any case, we are currently assessing the immunogenic potential of synthetic peptides derived from non-homologous regions (to its feline ortholog) of the *S. schenckii* enolase, which should eliminate any safety problems regarding the development of auto-immunity in the feline host.

Different studies have shown that enolase on the cell surface of bacteria, fungi and parasites acts as a virulence factor that facilitates the colonization and dissemination of these pathogens in the host^25,40,41^. Although there are no studies assessing the effects of the absence of enolase in *S. schenckii*, Ko *et al*.^42^ showed that *C. albicans ENO1* (enolase) null mutants exhibit reduced hyphal growth, decreased virulence in BALB/c mice and increased susceptibility to amphotericin B, miconazole and other antifungal drugs. We also know, from experiments of a yet to be published study from our group, that rSsEno is able to bind extracellular matrix constituents such as fibronectin and plasminogen and that an anti-rSsEno polyclonal serum generated in BALB/c mice inhibits the adhesion of *S. schenckii* ATCC 16345, *S. brasiliensis* Ss250, and *S. brasiliensis* Ss256 yeasts to fibroblasts, suggesting that enolase may be a virulence factor within the *Sporothrix* genus (data not shown). In this study, we show for the first time that enolase is present on the cellular surface of *S. schenckii* and *S. brasiliensis* species, and interestingly, this expression was higher on yeast cell walls from *S. brasiliensis*, suggesting that the level of enolase expression on the cell surface of species of the genus *Sporothrix* can be related to the invasiveness and virulence of these pathogens in the host. In this way, Roth *et al*.^43^ showed that the level of expression of enolase is 15-fold higher in red blood cells infected with *P. falciparum* compared to uninfected cells. More recently, Marcos *et al*.^44^ observed a considerable increase of this protein in the cell wall of *Paracoccidiodes brasiliensis* when the fungus was cultivated in BHI medium enriched with sheep blood or during fungal infection in mice, suggesting a role for enolase as a virulence factor of these fungi in host cells.

The generation of a Th1 and Th17 response is necessary for protective immunity against *Staphylococcus aureus* and *C. albicans*^45^. Ferreira *et al*.^46^ demonstrated in a model of *S. schenckii* infection in BALB/c mice that the Th1 and Th17 immune response were able to control the infection. Recently, our group reported a similar result in a model of C57BL6 mice subcutaneously infected with either *S. schenckii* or *S. brasiliensis*. However, the higher virulence of *S. brasiliensis* caused a long-lasting infection associated with severe tissue lesions that stimulated a regulatory T cell (Tregs) response with deleterious effects on the Th1 and Th1/Th17 response, although a compensatory Th17 response was induced^47^. We also demonstrated in an immunoprophylaxis study in BALB/c mice that either aluminum hydroxide adjuvant or PGA, both formulated with the *S. schenckii* ATCC 16345 CWPs containing the immunoreactive enolase, induced a Th1, Th2 and Th17 profile, in addition to high stimulation of specific antibodies that conferred protection in these animals after challenge with *S. schenckii* ATCC 16345 or *S. brasiliensis* Ss250^20^.

To verify whether rSsEno could be used as an antigenic target for a sporotrichosis vaccine, we performed a survival study in immunized mice after intravenous infection with the highly virulent strain *S. brasiliensis* Ss250. The survival above 90% seen in mice immunized with PGA + rSsEno100 is strong evidence of the protective capacity of our vaccine candidate. In addition, probably the Th1, and not Th1/Th2cytokine profile observed *ex vivo* in PGA + rSsEno100-immunized mice played a significant role *in vivo* in favoring protection, since rSsEno100-immunized mice showed *ex vivo* a stimulation of Th2 cytokines, which may be associated to decreased survival of those animals (~ 48%) postchallenge. Li *et al*.^24^ showed that a Th1 and Th2 immune response pattern induced by recombinant enolase of *C. albicans* emulsified with Freund’s adjuvant (AF) was enough to confer protection on C57BL/6 mice challenged with a lethal dose of *C. albicans* strains SC5314 and 3630. In addition, passive immune serum transfer, characterized by the prevalence of IgG2a- and IgG1-specific antigen isotypes, also demonstrated effective protection against both fungal *C. albicans* lineages, showing that antibodies against enolase could be useful to treat of candidiasis. Zhang *et al*.^48^ also showed that the enolase of *Streptococcus suis* serotype 2 plus AF formulation induced a mixed Th1 (IgG2a) and Th2 (IgG1) response that also conferred protection in challenged animals with two pathogenic strains of *S. suis*. This same immune response profile and protective efficacy were observed in mice immunized with the *Ascaris suum* enolase after infection with infective larvae of this parasite^49^.

Several studies have shown that the IgG antibody response^50,51^ and especially IgG1^52,53^, IgG2a and IgG3 isotypes^19,20^ against *S. schenckii* and *S. brasiliensis* cell wall proteins is associated with *in vivo* protection through neutralization (IgG1 and IgG3) and Fc-mediated phagocytosis by macrophages (IgG2a). Our results showed that rSsEno100 and PGA + rSsEno100 stimulated a Th2 (IgG1 and IgG3) and Th1/Th2 (IgG1, IgG2a and IgG3) immune response, respectively. Thus, the anti-*S. brasiliensis* protection conferred by the active or passive immunization of mice with anti-rSsEno may be related to the IgG profile. Moreover, Almeida *et al*.^54^ showed that opsonization with a humanized anti-gp70 (*Sporothrix* spp. immunogenic protein) IgG1 antibody (P6E7) increased the phagocytosis of *S. schenckii* yeasts by human monocyte-derived macrophages and that the passive transference of P6E7 to BALB/c mice three days post-infection decreased the fungal burden in the spleen a week later compared with the control. Therefore, a serotherapy-based strategy with anti-rSsEno holds great promise for the treatment and/or prevention of sporotrichosis.

In summary, for the first time, a recombinant form of *S. schenckii* (rSsEno) enolase was obtained folded and partially characterized. The weight-average molecular mass of rSsEno determined by SAXS was of about 580 kDa, while the aSEC showed a tail after the main peak indicating the presence of smaller oligomers. This organization is different from the enolases from other fungi, at least in the absence of magnesium which are enolase co-factors. The identification of enolase on the cell wall of *S. brasiliensis* and *S. schenckii* and its recognition by serum from cats affected with sporotrichosis are reported in this study. A vaccine formulation of rSsEno plus PGA adjuvant induced a Th1/Th2 response and high titers of specific antibodies that favored the protection to mice challenged with a highly virulent *S. brasiliensis* isolate. In addition, the anti-enolase serum induced by the vaccine candidate conferred protection to naïve mice. All these results show that the enolase of *Sporothrix* spp. may be a vaccine antigen candidate for feline sporotrichosis prevention.

## Materials and Methods

### Animals

For this study, male 5–7-week-old BALB/c mice were purchased from “Centro Multidisciplinar para Investigação Biológica na Área da Ciência de Animais de Laboratório” (CEMIB), Universidade de Campinas (UNICAMP), São Paulo, Brasil. Animals were housed in individually ventilated cages in an ambient controlled temperature and 12-h light/dark cycles. All animals were acclimatized to the conditions for 1 week before the experiments, and water and food was offered ad libitum. This study was carried out in strict accordance with the recommendations for the Guide for the Care and Use of Laboratory Animals of the National Institutes of Health, and the protocols were approved by the Institutional Ethics Committee for Animal Use in Research of the Faculty of Pharmaceutical Sciences of Araraquara – UNESP (Proc. CEUA/FCF/CAR no. 57/2015).

### Microorganisms

The strains *S. schenckii* ATCC 16345, *S. schenckii* 1099–18, *S. brasiliensis* Ss250 (GenBank: KC693883.1) and *S. brasiliensis* Ss256 (KC693889.1), both *S. brasiliensis* strains isolated from feline sporotrichosis, and *S. schenckii* ATCC 16345 were kindly provided by the Oswaldo Cruz Foundation, Rio de Janeiro, Brazil. *S. schenckii* 1099–18 was provided by Dr. Celuta Sales Alviano at the Institute of Microbiology, Federal University of Rio de Janeiro (Brazil). Mycelial-to-yeast phase conversion was accomplished as previously described by Ferreira and collaborators^46^.

### Expression and purification of recombinant *S. schenckii* enolase (rSsEno)

The encoding DNA for *S. schenckii* 58251 enolase containing 438 amino acids and a molecular mass of 47 kDa (Accession Code: ERS97971.1 of the GenBank database) was synthesized by Epoch Life Science Inc. optimized for production in *E. coli*. It was subcloned into the pET28a plasmid between the *Nde I* and *Eco RI* restriction enzymes in fusion with a His-tag at the N-terminus. The resultant plasmid (pET28a::SsEno) is capable of expressing a His-tagged rSsEno of about 50 kDa.

*Escherichia coli* DH5α was used as the cloning host for the propagation of pET28a::SsEno on lysogeny broth (LB) agar medium containing 30 μg/mL of kanamycin, and the authenticity of the cloning procedure was confirmed by sequencing. For recombinant protein expression, *E. coli* BL21 cells transformed with pET28a::SsEno were grown at 37 °C in LB medium containing 30 μg/mL of kanamycin until they reached an OD_600_ in the range of 0.5–0.7. The expression of rSsEno was induced by 0.2 mmol/L of isopropyl β-D-1-thiogalactopyranoside (IPTG) at 30 °C for 4 h. The cells were separated by centrifugation for 20 min at 8000 rpm, and the pellet was resuspended in 20 mL buffer A (NaPO_4_ 20 mM, NaCl 500 mM and imidazole 20 mM, pH 7,4) containing 5 U of DNAse (Promega) and 30 ug/mL lysozyme (Sigma) for 30 min on ice. The cell homogenate was sonicated, filtrate and then centrifuged at 19,000 rpm for 20 min at 4 °C. The rSsEno-containing supernatant was filtered through a Hydrophlic Durapore Membrane, 0.45 µm cutoff, 47 mm diameter (Millipore) and further subjected to Ni^2+^-affinity chromatography in buffer A. The rSsEno was then eluted in buffer B (NaPO_4_ 20 mM, NaCl 500 mM and imidazole 500 mM, pH 7,4). After elution, the material obtained was subjected to a preparative SEC with a Superdex 200 pg 16/60 column (GE Healthcare Life Sciences) in Tris-HCl 25 mM, NaCl 100 mM and β-mercaptoethanol 2 mM at pH 7.5, and subjected to biophysical tests. The eluted protein was also concentrated using the *Amicon Ultra 15 mL 3k* device (Millipore) after being dialyzed for 24 h at 4 °C against phosphate buffer saline (PBS, pH 7, 2–7, 4). The rSsEno concentration was measured by the BCA assay (Pierce) (for immunological tests) or by absorbance at 280 nm (for biophysical tests) using an extinction coefficient of 54,620 M^−1^ cm^−1^, obtained from the rSsEno primary structure through Protparam Tool (web.expasy.org/protparam/). The efficacy of the expression and purification processes was assessed by 12% SDS-polyacrylamide gel electrophoresis (SDS-PAGE).

### Biophysical characterization

Secondary structure analysis for rSsEno was performed by far-UV (195–260 nm) CD in a J-815 spectropolarimeter (Jasco Inc.) coupled to a Peltier PFD 425 S for the temperature control system. rSsEno was tested in Tris-HCl buffer (pH 7.5), 100 mM NaCl and 2 mM β-mercaptoethanol, at 20 °C and the secondary structure content was estimated using the CDNN Deconvolution program^55^. The folded structure for rSsEno was also investigated by intrinsic fluorescence emission using a Fluorescence Spectrophotometer Hitachi F-4500 with excitation wavelength at 295 nm and recording the emission fluorescence spectra from 305 nm to 420 nm. The recombinant protein was prepared at 1 µM in Tris-HCl buffer (pH 7.5), 100 mM NaCl and 2 mM β-mercaptoethanol, at 20 °C. The effect of a chemical denaturant, 6 M guanidinium-HCl (Gnd-HCl), on the rSsEno structure was also investigated by intrinsic fluorescence.

In addition, the rSsEno oligomeric state was analyzed by analytical size exclusion chromatography on a Superdex 200 GL 10/30 column (GE Healthcare LifeSciences) coupled to a ÄKTA Prime Plus (GE Healthcare LifeSciences) and equilibrated with the same buffer described above, at room temperature. Blue dextran was used in order to identify the column void. The column was calibrated using a pool of protein markers of known size.

The rSsEno oligomeric state was also investigated by small angle X-ray scattering (at SAXS1 beamline, at the Brazilian Synchrotron Light Laboratory, Campinas, Brazil). Measurements were done using a monochromatic X-ray beam (λ = 1.488 Å), and rSsEno at 0.5 and 0.9 mg.mL^−1^ (prepared in Tris-HCl buffer pH 7.5, 100 mM NaCl and 2 mM β-mercaptoethanol) were applied into a 1 mm path length capillary mica. Data were recorded using a Pilatus 300 K detector, with a ~900 mm sample-to-detector distance. Scattering curves were normalized by concentration, extrapolated to infinite dilution and used to yield the Guinier curve in order to estimate-average molecular mass and the average size of the scattering particle^56,57^.

### Extraction of *S. schenckii* ATCC 16345 CWPs

Extraction of the *S. schenckii* ATCC 16345 CWPs was performed per Portuondo *et al*.^19^. *S. schenckii* ATCC 16345 yeast cells collected from logarithmically growing cultures were incubated with a protein extraction buffer containing 2 mM dithiothreitol, 1 mM phenylmethylsulfonyl fluoride, and 5 mM EDTA in This-HCl buffer for 2 h at 4 °C under mild agitation. The *S. schenckii* ATCC 16345 CWPs-containing supernatant was collected, dialyzed against PBS, and then concentrated using the *Amicon Ultra 15 mL 3 k device* concentrator (Millipore). The proteins were then precipitated by overnight incubation with 10% (w/v) trichloroacetic acid in acetone at 4 °C, and the resulting pellets were washed in ice-cold acetone, dried in a SpeedVac and reconstituted PBS. The protein concentration was measured by the BCA assay (Pierce).

### SDS-PAGE, western blot analysis

Samples containing 20 µg of protein *S. schenckii* ATCC 16345 CWPs and purified rSsEno (5 µg) were resolved on an SDS-PAGE 12% as described by Laemmli^58^. Two gels were stained with Coomassie brilliant blue R250, and the other gels were transferred to 0.45-μm-nitrocellulose membranes (GE Healthcare) using a mini Tank VEP-2 electroblotting system (Owl Separation Systems, Thermo Scientific) at 50 mM for 3 h. The membrane-cut strips were saturated with 5% dried skim milk in PBS for 4 h at 37 °C, and the strips containing rSsEno were incubated overnight at room temperature (RT) with anti-rSsEno serum (obtained from BALB/c mice seven days after being immunized subcutaneously twice at 14 day intervals with 100 µg rSsEno emulsified with Freund´s adjuvant) or sera from cats with confirmed sporotrichosis* (n = 34) obtained from the Laboratório de Pesquisa Clínica em Dermatozoonoses em Animais Domésticos (Lapclin-Dermzoo)/Instituto Nacional de Infectologia Evandro Chagas (INI)/Fundação Oswaldo Cruz, Rio de Janeiro, Brazil. One strip containing *S. schenckii* ATCC 16345 CWPs was incubated with anti-rSsEno. Sera from naïve mice or sera from cats with no evidence of sporotrichosis (n = 3) were utilized as negative controls. All sera were diluted 1:100 in PBS. After three washes with PBS, the strips were further incubated for 2 h with goat anti-mouse IgG (Sigma-Aldrich) diluted 1:500 or goat anti-feline IgG (Southern Biotech) diluted 1:1000. Both antibodies were conjugated with horseradish peroxidase (HRP). Protein signals were visualized by adding 3,3′-diaminobenzidine *plus* hydrogen peroxide. *The sporotrichosis diagnosis was carried out as follow: Samples from skin or nasal mucosa lesions were collected using a sterile swab and seeded onto Sabouraud Dextrose Agar and Mycobiotic Agar (Difco), incubated at 25 °C for four weeks. Microscopic and macroscopic characteristics of the mycelia cultures were evaluated on Potato Dextrose Agar. Dimorphism was confirmed by conversion to the yeast phase on BHI Agar at 37 °C.

### Alignment of enolase sequences

We compared conservation (similarity) between the enolase of *S. schenckii* and the cat and human enolase. The enolase amino acid sequences of *S. schenckii* (GenBank Accession No. ERS97971.1 and *Felis catus* (UniProt Accession: M3 WCP0_FELCA, Homo sapiens (UniProt Accession: P06733) were aligned through the default settings within Clustal Omega^59^.

### Flow cytometry

To demonstrate the enolase on the cell wall of *S. schenckii* ATCC 16345, *S. schenckii* 1099–18, *S. brasiliensis* Ss250 and *S. brasiliensis* Ss256, 10^6^ yeasts were incubated for 1 h at 37 °C with anti-rSsEno serum. Serum from naïve mice was used as a nonspecific binding control at a 1:50 dilution. After incubation, cells were washed twice with PBS for 1 h at 37 °C and then incubated with a FITC-conjugated rabbit anti-mouse IgG antibody (Sigma-Aldrich) at a 1:500 dilution. After washing, samples were acquired with the BD Accuri C6 flow cytometer (BD Biosciences). The acquisition threshold was set to 50,000 on FSC-H for debris exclusion, and at least 50,000 events were effectively included in each analysis. Binding of serum antibodies to the yeast cell surface was assessed through the median fluorescence intensity (MFI) on the FL1 channel using the flow cytometer’s proprietary software.

### Electron microscopy

To visualize enolase on the *S. schenckii* ATCC 16345, *S. schenckii* 1099–18, *S. brasiliensis* Ss250, and *S. brasiliensis* Ss256 cell surface, we performed pre-embedding immunogold experiments using intact yeast cells this fungus, as described previously^43^. Briefly, the yeast cells were fixed with 2.5 glutaraldehyde v/v in 0.1 M cacodylate buffer, pH 7.2, for 24 h at 4 °C. Ultrathin sections of each fungus were prepared and treated overnight with the primary antibody (polyclonal anti-rSsEno) diluted 1:100 in PBS at 4 °C. The grids were then incubated overnight with the labeled Au-conjugated secondary antibody rabbit IgG (10 nm average particle size, 1:20) at 4 °C. The grids were stained with 4% uranyl acetate and lead citrate and observed with a Jeol 1011 transmission electron microscope (Jeol, Tokyo, Japan). Controls were obtained by incubating the ultrathin sections with NS.

### Immunization schedule

BALB/c mice (n = 5) were injected subcutaneously (s.c.) three times in the back of the neck, with 2-week intervening period, with one of the following formulations diluted in 100 μl of PBS: rSsEno100 alone (100 μg), PGA + rSsEno100 [10% Montanide Pet Gel A (PGA), SEPPIC, France plus 100 μgrSsEno] or PBS alone as a negative control. One week after the lastimmunization, mice were euthanized in a CO_2_ chamber and bled by heart puncture to obtain serum, which was aliquoted and stored at −20 °C until use.

### Quantification of the rSsEno-specific antibody response by ELISA

rSsEno IgG, IgG1, IgG2a and IgG3 antibody titration was conducted as described by Portuondo *et al*.^19^ with some modifications. Briefly, a 96-well ELISA plate (Costar) was coated with 5 μg rSsEno/mL in PBS and incubated overnight at 4 °C. The plate was washed with washing buffer (0.1% Tween 20 in PBS) and then saturated for 1 h at RT with blocking buffer (5% dried skim milk in washing buffer). Next, dilutions (1:100 in blocking buffer) of the serum samples were added to each well and incubated for 2 h at RT. After washing, 100 µl/well of peroxidase-conjugated anti-mouse IgG (1/500) (Sigma) in blocking buffer was added and incubated at 37 °C for 1 h. For determination of the IgG1, IgG2a and IgG3 subclasses, ELISA plates coated as before were first incubated with an unconjugated rabbit anti-mouse IgG1, IgG2a or IgG3 (Bio-Rad) at 37 °C for 1 h and then with a peroxidase-conjugated goat anti-rabbit IgG (Sigma) overnight at 4 °C. After exhaustive washing, immune complexes were revealed by incubation with tetramethylbenzidine for 30 min at RT. The reaction was stopped by the addition of 50 µL/well 1 M H_2_SO_4_, and the absorbance was read with an ELISA reader (Multiskan ascent, Labsystem) at 450 nm.

### Cytokine production

To evaluate the cytokine production induced by rSsEno-stimulated spleen cells, splenocytes isolated from each group of animals were harvested seven days after the third immunization. Collected cells were washed, suspended in complete RPMI−1640 medium (cRPMI; RPMI−1640 medium containing 0.02 mM 2-mercaptoethanol, 100 U/mL penicillin, 100 U/mL streptomycin, 2 mM l-glutamine, and 5% fetal bovine serum) and then plated in triplicate in 96-well plates (Costar, USA) to final concentration 2.5 × 10^6^ cells/mL with 20 µg of rSsEno/mL in cRPMI for 24 h at 37 °C with 5% CO_2_. Concanavalin A (0.25 l g/ml) or cRPMI alone were used as positive and negative controls, respectively. Supernatant-accumulated cytokine concentrations (IL-2, IL−10, IL-4, IL-6, IFN-γ, TNF-α, and IL-17A) were simultaneously measured using the mouse Th1/Th2/Th17 cytokine cytometric bead array (CBA) kit (BD Biosciences). Briefly, 50 μL of each standard or supernatant sample was incubated for 2 h at RT with an equal volume of PhycoErythrin (detection reagent) and the mixed capture beads. After incubation, the samples were centrifuged at 200 × g for 5 min, and the pellet was resuspended in 300 μL of wash buffer and analyzed using a flow cytometer (BD Accuri C6, BD Biosciences).

### Protection assay

BALB/c mice (n = 10) were immunized according to the immunization schedule described previously. Seven days after the final boost, mice were challenged intravenously with 10^5^ of the highly virulent *S. brasiliensis* Ss250 yeasts in 0.1 mL of PBS via the tail vein, as described by Ishida *et al*.^60^. Animals were monitored daily for 45 days postinfection to determine the survival curve and efficacy of each vaccine formulation. In another experiment, a 200 µL of a 1:2 dilution of a pooled immune serum from PGA + rSsEno100-immunized mice was passively transferred via the i.p. route to BALB/c mice (n = 7) two hours prior to infection with 10^6^ *S. brasiliensis* Ss250 yeasts also via the i.p; mice pre-treated with NIS or PBS were used as controls. Protection was assessed by determining the number of CFUs recovered from the spleen and liver on day 5 post-infection. This was done by plating an adequate dilution of the organs’ macerate on Mycosel agar plates.

### Statistical analysis

All statistics were performed using Graph Pad Prism version 6.01 by applying Student’s t-test or one-way analysis of variance (ANOVA) followed by Tukey’s post-test. Survival data were compared using the log-rank test. In this study, a p value of < 0.05 was considered significant. The results are expressed as the mean ± SD.
